# Valedictory Address to the Graduating Class, Ohio Dental College, March 3d, 1869

**Published:** 1869-03

**Authors:** E. Rives


### VALEDICTORY ADDRESS TO THE GRADUATING CLASS, OHIO DENTAL COLLEGE, MARCH 3, 1869.

BY PROF. E. RIVES.

When those with whom we have been pleasantly and profitably associated for a length of time are about to depart from our midst, and when we reflect that the natural changes and vicissitudes of life make it improbable that we will all meet together again, it becomes a painful duty to say "Good-bye."

But when those who leave us are of the same household of faith, working in the same cause, having the same aspirations to relieve human suffering, and have, by diligent study, made themselves competent to commence their professional journey through life, the natural sorrow at parting is softened, because those who have identity of interests and aspirations can hardly be said to be separated, even though oceans intervene. In spirit we will ever be united.

And then when we look on your familiar faces, radiant with the glow of success, full of bright anticipations of the future, expressive of eagerness to engage in the great battle of life, it would be cruel to dim one bright thought by uttering words of sorrow at parting.

No! Our good-bye must be cheery, for all your high hopes, joyous anticipations and firm resolves find a responsive echo in our hearts, and while we deeply regret that our pleasant association has come to an end, (at least in our former relation) yet we rejoice that the noble army of engineers on the road to truth has been increased by so worthy an addition, and we bid you go forth in the name of truth and science, not in sorrow, but with joyous expectations of your success.

On such occasions, I believe, it is customary to accompany the presentation of diplomas with a large quantity of advice, both of a professional and moral nature. This, doubtless, is a good old custom and sanctioned by years of habit, and I

dare not condemn it, but you will pardon me if I decline undertaking the moral advice which belongs more properly to your spiritual adviser. I feel, too, a hesitancy in assuming the character of a *mentor* to graduates in the Ohio College of Dental Surgery.

It occurs to me that the diplomas which you hold (or are about to receive) are sufficient guarantee that you have received and accepted the advice of the several professors in this institution.

We believe that you are in the right road and will be able to find your way, if you only hold in remembrance the cardinal maxim taught within these walls, namely: "Be students always," and never forget that to stand still in life is impossible—you *must* advance or you *must* lose ground. This brings me to the contemplation of a point upon which I hope you will indulge me in dwelling for a few minutes, because it particularly interests you. It relates to *work*.

We hear, occasionally, of men killing themselves by work. The instances must be exceedingly rare. The healthiest men I know of are the hardest workers, and I do not remember a single instance in my personal experience where work has *injured*, much less killed any one. You will generally find that the reported cases of death from over-work, when investigated, have resulted from too little work, injudicious work, or some more flagrant violation of nature's laws. Walk Fourth street on a bright afternoon and see the miserable specimens of manhood dying from *idleness* and consequent inanity, lounging around the corners until they are pushed out of the way by policemen. These men, gay and careless as they seem, are dying because they do not work. See, on the same street, the thrifty working man (either of brain or body) passing these idlers, with bright eye and elastic step, full of good resolves and high motives, and tell me which promises the longest life. O no! Work never injures. It is idleness which is the canker worm that eats out men's souls. But then a man may work injudiciously, and yet work very hard. I mean by this, that he may confine himself too much to one idea, and thereby wear out those faculties of mind or body engaged in the development of that idea. Confining ones mind continuously to one idea dwarfs the intelligence (as confining one set of muscles only to the accomplishment of one object, atrophies those muscles in disuse.)

We have been mercifully endowed by the Almighty with wonderful faculties, both mental and physical, not to keep as a miser does his gold, but to improve and to increase in power. This you can not accomplish by the exercise of one or a few of those faculties, to the exclusion of others. You must apply them all, physical and mental, by turns and together, in various combinations, if you expect to attain to any degree of human perfection.

Success in life depends largely on the degree of perfection to which our mental and physical natures have arrived. This degree of perfection depends upon the exercise of every faculty with which we are endowed. In a word, keep none of your faculties of mind or body in disuse. You will find time and occasion to use them all, either in connection with or having some relation to your legitimate business. An harmonious development of all your faculties will assist, rather than retard you in the grand object of your life.

Temperance should be observed in the use of our faculties, as it should be in the use of the cup. You may be intemperate in the use of either. True work then, is the judicious and temperate use of every faculty we possess, by turns or in combination.

I will detain you on another point, and will state it as follows:

No one can justify himself, however high his position and great his fame, in condemning, without patient investigation, any opinion or proposition expressed by an earnest, honest man, even though at first it may seem absurd. I speak of this, because if we look back into the history of science we find numerous instances where the grandest discoveries were met with scoffs and jeers. One instance in point: The great Copernicus first announced the startling fact that our planetary system revolved around the sun not only, but that all other systems revolved around their suns, and that each particular orb revolved on its own axis. These great truths have given rise to the grandest and most important discoveries, and yet this master mind, able to grasp so stupendous an idea, was adjudged insane by many of his most illustrious cotemporaries, and was in fear and danger of persecution.

Again: With what difficulty, and after how many years of patient trial, meeting with scoffs and ridicule, did Columbus succeed in obtaining means to verify his "crazy" idea of another continent. Yet here we are firmly established on the "crazy idea," in the shape of the continent of North America, to which the world is rushing in "crazy" speed.

This is only to illustrate the care with which we should condemn any scientific proposition. While on the one hand we should be slow to condemn, on the other we should be slow to accept. Wait and investigate. Base your rejection or acceptance on well known facts. Never have a pet idea. All true ideas should be your pets. In a word, endeavor to weigh all propositions without bias on either side.

The examination through which you have just successfully passed, and which doubtless caused you much anxious solicitude, you will be astonished to hear, is not the last ordeal of the kind—through which you must pass. Do not be alarmed! This Faculty has awarded you your diplomas. I mean that every day of your life you will be subjected to the silent, but, in the main, just examination of a discriminating public. And more, every night will be repeated the most unrelenting of all examinations, namely: that of your own consciences. These you can never expect to escape—go where you will—do what you may. You must ever keep yourself in a state of preparation for examinations. They increase in difficulty as we grow older, and we must ever be "up and doing," if we hope to succeed.

We hope and believe that each of you will be as successful in these examinations as in the ones through which you have just passed.

Upon reflection, I doubt very much whether I should fly in the face of custom, and say nothing to you of a "moral" nature. To save my own conscience, I will simply say what your kind, good parents no doubt told you when little boys, and in a far more impressive and emphatic way, "Be good and true." These attributes should attach not only to little boys, but to grown up men, and even to Dental graduates.

